# Gastrointestinal cancers in China, the USA, and Europe

**DOI:** 10.1093/gastro/goab010

**Published:** 2021-03-29

**Authors:** Yumo Xie, Lishuo Shi, Xiaosheng He, Yanxin Luo

**Affiliations:** 1 Department of Colorectal Surgery, The Sixth Affiliated Hospital, Sun Yat-sen University, Guangzhou, Guangdong, China; 2 Guangdong Institute of Gastroenterology, Guangdong Provincial Key Laboratory of Colorectal and Pelvic Floor Disease, The Sixth Affiliated Hospital, Sun Yat-sen University, Guangzhou, Guangdong, China; 3 Center for Clinical Research, The Sixth Affiliated Hospital, Sun Yat-sen University, Guangzhou, Guangdong, China

**Keywords:** colorectal cancer, gastric cancer, esophageal cancer, epidemiology, cancer screening

## Abstract

Gastrointestinal (GI) cancers, including colorectal cancer, gastric cancer, and esophageal cancer, are a major medical and economic burden worldwide and have the largest number of new cancer cases and cancer deaths each year. Esophageal and gastric cancers are most common in developing countries, while colorectal cancer forms the major GI malignancy in Western countries. However, a great shift in the predominant GI-cancer type is happening in countries under economically transitioning and, at the same time, esophageal and gastric cancers are reigniting in Western countries due to the higher exposure to certain risk factors. The development of all GI cancers is highly associated with lifestyle habits and all can be detected by identified precancerous diseases. Thus, they are all suitable for cancer screening. Here, we review the epidemiological status of GI cancers in China, the USA, and Europe; the major risk factors and their distribution in these regions; and the current screening strategies.

## Introduction

Cancer is the first or second leading cause of premature death (at ages 30–69 years) in most countries, and premature death caused by cancer accounted for 29.8% of deaths from non-communicable diseases with a total of 4.5 million in 2016 [1]. In 2020, it was estimated that there were 19.3 million new cancer cases and 9.9 million cancer deaths, rising from 14.1 and 8.2 in 2018 to 18.1 and 9.6 in 2019 [2–4]. Due to the growth and aging of the population and the inequality in cancer control, cancer has become more prominent as a cause of death and challenges the previously predominant ischemic cardiovascular diseases [5]. The burden of cancer is expected to grow worldwide, particularly in less developed countries [6].

Gastrointestinal (GI) cancers, mainly including the malignancies derived from esophageal, stomach, and colorectum, are among the most common cancers in humans. These cancers, which are derived from distinct but associated origins, have diverse clinical features but share some similar characteristics. According to the data available from GLOBOCAN 2020, GI cancers [colorectal cancer (CRC), gastric cancer, and esophageal cancer] accounted for 18.7% of new cancer cases and 22.6% of cancer deaths in 2020, which are both highest among all cancer types, and are a significant public health burden for most countries [4]. However, in terms of the geographic and temporal distribution, major risk factors, and prevention strategies of GI cancers, great differences exist between the West and the East. In this article, we review the epidemiological data from China, Europe, and the USA; risk factors; and current progress in the prevention and screening of GI cancers in these and other countries.

### CRC

CRC is the third most commonly diagnosed malignancy in males and the second in females, and the second leading cause of cancer death in both sexes [3]. CRC comprises 10% (1.9 million) of global new cancer cases and 9.4% (0.9 million) of cancer deaths in 2020 [4]. The global disease burden in 2016 was estimated as 17.2 million disability-adjusted life years, of which 97% came from years of life lost due to premature mortality and 3% came from years of healthy life lost due to disability [1].

### Epidemiology characteristics

**Figure 1.**
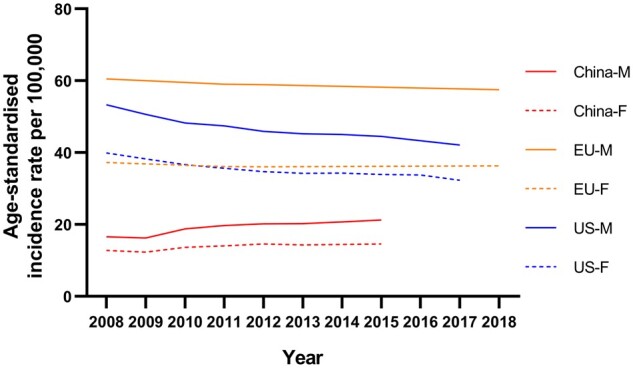
Time trends of incidence rates of colorectal cancer in men and women across China, the USA, and Europe. Data from National Cancer Center (China), Centers for Disease Control and Prevention (the USA), and European Cancer Information System (EU).

The incidence of CRC is unbalanced throughout the world and varies greatly between high and low human development index (HDI) regions. The incidence is about 3-fold higher in high-HDI regions vs low-HDI regions, but the average case fatality is higher in low-HDI regions. Furthermore, there are also rapid rises in incidence in countries undergoing economic development and changes in diet and lifestyle [7]. The incidence of CRC exhibits a preference in populations with ‘Western’ lifestyles brought by industrialization and economic growth, since diet, physical activities, and obesity are major factors associated with CRC [8]. This pattern can be observed in the USA and Europe, which have both high HDI and high age-standardized incidence rates (ASRs) [9, 10]. Besides, in those countries that are economically transitioning, like China, the incidence rates of CRC are under rapid growth (Figure 1). In China, CRC was the fourth most common cancer type at the beginning of the twenty-first century [11]. However, as reported by the National Cancer Center, the incidence rate of CRC ranked third (ASR 17.8 per 100,000) among all cancer types in 2015 [12]. Furthermore, among the three areas (Eastern, Middle, Western), CRC was the second common cancer type in the Eastern area of China (19.9 per 100,000), which stands for the most developed region of China [11, 12]. Moreover, it has been estimated by GLOBOCAN to have been the second most prevalent cancer in 2020. In 2020, as estimated by GLOBOCAN, China had the largest number of CRC cases, while the USA ranked second. The incidence rate in China is close to that in the USA (23.9 vs 25.6 per 100,000), especially in males (28.6 vs 28.7 per 100,000), whereas the incidence rate in Europe was still way ahead (30.4 per 100,000) (Table 1).

**Table 1.** The estimated incidence and mortality (per 100,000) of gastrointestinal cancers by age groups in GLOBOCAN 2020

	China			Europe			USA		
	Total	Male	Female	Total	Male	Female	Total	Male	Female
All ages (ASRW^a^)									
Colorectal cancer									
Incidence	23.9	28.6	19.5	30.4	37.9	24.6	25.6	28.7	22.9
Mortality	12.0	14.8	9.4	12.3	16.1	9.5	8.0	9.4	6.7
Gastric cancer									
Incidence	20.6	29.5	12.3	8.1	11.5	5.3	4.2	5.3	3.1
Mortality	15.9	22.8	9.5	5.5	7.9	3.5	1.7	2.2	1.3
Esophageal cancer									
Incidence	13.8	19.7	8.2	3.3	5.8	1.3	2.8	4.8	1.1
Mortality	12.7	18.3	7.4	2.7	4.9	1.0	2.4	4.2	0.8
≥50 years									
Colorectal cancer									
Incidence	106.4	125.0	88.4	166.5	203.5	136.8	117.2	131.3	104.6
Mortality	56.8	66.6	47.3	80.3	97.2	66.8	42.7	47.8	38.2
Gastric cancer									
Incidence	92.1	131.3	54.2	43.1	59.8	29.7	20.2	26.3	14.7
Mortality	73.9	104.0	44.7	31.0	42.8	21.5	8.8	11.3	6.6
Esophageal cancer									
Incidence	64.9	90.0	40.8	16.9	28.9	7.3	14.9	24.7	6.0
Mortality	61.0	84.6	38.1	14.7	25.2	6.3	13.3	22.5	5.0
<50 years^b^									
Colorectal cancer									
Incidence	7.8	8.4	7.2	8.8	8.5	9.1	12.8	12.7	12.9
Mortality	2.5	2.8	2.2	2.3	2.3	2.2	3.1	3.4	2.8
Gastric cancer									
Incidence	6.4	7.6	5.1	2.7	3.1	2.4	1.9	1.9	1.8
Mortality	3.6	4.1	3.0	1.7	1.9	1.6	0.8	0.8	0.7
Esophageal cancer									
Incidence	2.5	3.9	1.0	1.0	1.5	0.4	0.6	1.0	0.3
Mortality	1.8	2.8	0.7	0.6	1.1	0.2	0.4	0.7	0.2

a Age-standardized rate by world standard population (Segi’s population).

b Including people aged 20–49 years.

In some countries with very high HDI, the incidence rates of CRC are now experiencing a decreasing trend, such as in the USA (Figure 1), Australia, and Japan. Arnold *et al*. [13] identified three categories based on temporal characteristics of incidence and mortality: group 1: increasing incidence and mortality (China, Brazil, Russia, Spain, etc.); group 2: increasing incidence and decreasing mortality (Canada, Denmark, UK, Singapore, etc.); group 3: decreasing incidence and mortality (USA, Australia, New Zealand, Iceland, etc.). China, for example, had rapidly increasing new CRC cases and the mortality rate has also been rising during recent decades, from an ASR per 100,000 of 6.18 in 2008 to 8.12 in 2015 [12, 14]. Moreover, the mortality rate of CRC in China was estimated to have overtaken that in the USA in 2020 (ASR per 100,000: 12.0 vs 8.0) [4]. In the USA, according to the Centers for Disease Control and Prevention (CDC), the mortality rate of CRC has been decreasing since 1999, from 20.9 in 1999 to 13.5 in 2017 (ASR per 100,000) [9]. The decrease in mortality can be attributed to the improvements in survival through the adoption of best practices in cancer treatment and management and increased screening leading to early detection. The decreasing incidence rates in group 3 countries may be due to the delayed effect of the screening programs. The introduction of screening programs may have initially increased the incidence rates, as there was more detection of early-stage CRC, but this has been proven to have resulted in lower incidence rates because of the removal of precancerous lesions by endoscopy [15].

The incidence and modality of CRC both increase rapidly after the age of 50 years [16, 17]. According to GLOBOCAN, in 2020, >90% of CRC cases and deaths occurring after this age. Although most CRC patients were diagnosed at an older age, an increasing trend for incidence in younger populations is emerging. While early-onset CRC has a familial component more often than late-onset disease, most cases are sporadic [18]. In the USA, increasing risk and incidence of CRC in those between 20 and 49 years old were found in sequential birth cohorts [19]. The incidence increased from 6.4 per 100,000 in 2002 to 8.3 per 100,000 in 2017 and was estimated to have reached 12.8 in 2020 [4, 9]. The incidence rate of early-onset CRC for the USA is higher than that for Europe, where the highest all-age incidence rate of CRC can be found (Table 1). These strong birth cohort effects may signal relatively recent changes in exposures that influence risk.

Higher incidence and mortality can be observed in men (Table 1). This may relate to a series of complicated factors. Several studies have indicated that men are more vulnerable to environmental factors in developing CRC [20, 21], and men also have higher exposure rates to the risk factors for CRC, such as alcohol intake, smoking, and obesity [22–24]. Moreover, men are inherently not protected by estrogen, which was known to be inversely associated with colorectal-cancer risk [25].

### Risk factors

Genetic and environmental factors both play an important role in the etiology of CRC. Genetics contribute to individual risk, but environmental factors, including diet and lifestyle, affect the incidence in populations. About 75% of patients have a negative family history, suggesting that most CRCs are sporadic [20]. Several studies also indicated that, in the USA and Europe, ∼16%–71% of new CRC cases can be attributed to environmental factors [26, 27]. Thus, key behavior modifications and adherence to a healthy lifestyle could have avoided most CRC cases. Smoking is the most important lifestyle risk factor [28]. According to the continuous update project report by the World Cancer Research Fund/American Institute for Cancer Research (WCRF/AICR), obesity, low physical activity, Western diet habits, and alcohol increase CRC risk [8].

Regarding genetic factors, the two most common hereditary colorectal-cancer syndromes are Lynch syndrome (hereditary nonpolyposis CRC) and familial adenomatous polyposis (FAP), and they together account for 5%–10% of CRC patients [29]. Lynch syndrome comprises 2%–4% of CRC cases and is caused by a mutation in one of the DNA mismatch-repair genes: MLH1, MSH2, MSH6, PMS2 or EPCAM [29]. Lynch syndrome increases the lifetime risk by ≤60%. In this setting, cancers evolve relatively quickly from adenomas or possibly even normal-appearing tissue and frequently elicit strong immunological responses [30]. FAP accounts for <1% of CRC cases and is caused by the adenomatous polyposis coli gene. Patients usually develop many adenomas at a young age, mainly in the distal colon. If the adenomas are not removed adequately, the risk of CRC would add up to 100% by 40 years of age [31].

Adenomas and serrated polyps are two major subtypes that are precursors to the majority of sporadic CRCs [32]. Approximately 85%–90% of sporadic CRCs evolve from adenomas. Advanced adenomas (≥1 cm in diameter, villous histology, or high-grade dysplasia) with or without multiplicity (more than three adenomas) have a significantly higher rate (30%–50%) of progress to CRC. Serrated polyps represent a group of heterogeneous lesions, including hyperplastic polyps, traditional serrated adenomas, and sessile serrated adenomas [33]. Patients with sessile serrated adenoma or traditional serrated adenoma are at increased risk of CRC and some would propose that the risk is similar to or higher than that for patients with conventional adenomas. The odd ratios (ORs) of CRC were 3.40 (2.35–4.91) for sessile serrated adenomas, 4.84 (2.36–9.93) for traditional serrated adenomas, and 2.51 (2.25–2.80) for conventional adenomas, compared with individuals without a history of polyps [34].

Now it has been recognized that the consumption of Western-type calorically-rich diets combined with chronic overnutrition and a sedentary lifestyle in Western societies evokes a state of chronic metabolic inflammation, which contributes to the development of CRC and other diseases, and this situation is also becoming more and prevalent in China [35].

Obesity is recognized as an established risk factor for CRC, and its effects are stronger in colon cancer than in rectal cancer [36]. With each unit increase in body mass index (BMI), the risk of CRC increases by 2%–3%. Moreover, this linear association was stronger in North American than European populations, but not significant in the Asian population. However, Asian individuals had a sharply increased risk from BMI < 23 kg/m^2^ to a relatively normal range (23–25 kg/m^2^), as each 5-kg/m^2^ increment was associated with an 18% increased risk [37]. This phenomenon may due to the difference in body-fat distribution between the West and the East. Recent studies suggest that waist circumference as a stronger risk factor [38], due to rising evidence on the effects of abdominal/visceral fatness on CRC [39, 40]. Abdominal fatness is more common in Asians than in Caucasians [41], which might contribute to the elevated risk of CRC in the Asian population with normal BMI.

Physical activity is well known for its potential for reducing cancer risk, but it is only established in colon cancers as an evidential risk factor [42], and the evidence in rectal cancer is not significant. Exercise may render its benefits mainly through its effect on weight loss [43], while other studies have also illustrated its benefits through enhancing gut motility, IL-6 redistribution, epinephrine release, and activating the immune system against tumors [44].

Dietary habits, both the healthy pattern and the unhealthy pattern, are well recognized as an important factor in the etiology of CRC [8, 45]. Intake of red meat and processed meat increases the risk of CRC by an estimated 1.16-fold per 100-g increase in daily intake [46]. By contrast, the consumption of milk, whole grains, fresh fruits, and vegetables, as well as an intake of fiber, multivitamins, and vitamin D, decreases the risk of CRC [47]. Of note, fiber supplements failed to protect against recurrent colorectal adenomas [48], which might imply overestimation in fiber supplements and the potential benefits of natural grains. As a result, dietary fiber was demoted to ‘probable evidence’ in the 2018 version of the continuous update project for CRC [8].

For calcium intake, each 300-mg/day increase was associated with an ∼9% reduced risk of CRC in a large observational study [49]. But randomized control trials failed to prove this finding with calcium supplements, even together with vitamin D [50, 51]. This may due to the calcium calculated in observational studies being mostly from dairy products and varying over a wider range. Nevertheless, calcium is considered to have potential against the development of CRC but, for now, the evidence is not sufficient to recommend calcium supplements for an anti-CRC purpose.

Moderate alcohol consumption (50 g per day) has been estimated to increase CRC risk by 38%, whereas even higher alcohol consumption (100 g per day) is associated with an ≤82% increased risk, and the association was stronger in Asians [52]. Cigarette smoking is significantly associated with CRC incidence and mortality. Smoking leads to a risk increase of 18% and an increase of 10.8 new cases per 100,000 person-years [53]. Interestingly, recent studies have shown that smoking is differentially associated with the risk of different molecular subtypes. Ever smoking was associated with microsatellite instability (MSI)-high and microsatellite stability (MSS)/MSI-low CRC, but the association was significantly stronger for ∼50% for MSI-high CRC [54], and cigarette smoking was also associated with the CIMP-positive and BRAF mutation-positive colorectal-cancer subtypes [55].

Inflammatory bowel disease (IBD) is also associated with an increased risk of CRC and the risk is higher when the history of IBD is longer [56]. However, it only explains ∼1% of all CRC in Western populations. Moreover, its risk of CRC seems to decreases over time due to the improved therapies for patients with IBD [57].

### Prevention and screening

In addition to changing bad lifestyles, the application of aspirin has been extensively examined for its potential against CRC [7]. Aspirin was reported to protect patients from adenoma recurrence and the development of CRC [58, 59]. A long-term daily aspirin could reduce ∼25% incidence and 39% mortality. It was estimated that a minimum intake of 325 mg aspirin per week for ≥6 years might be required to obtain any benefit against CRC [60].

CRC is one of the cancers that can benefit most from screening, since the grossly visible lesions usually take 10 years to progress to CRC, leaving a wide time window for early diagnosis [61]. Screening can help to remove the precancerous lesions by colonoscopy or diagnose cancer at an earlier stage, which will reduce the incidence and mortality, and thus lower the burden of CRC. CRC is more suitable for population screening than any other malignancy owing to a combination of factors [62]. Major approaches for screening include a fecal occult blood test (FOBT), colonoscopy, and other tests based on stool samples.

The FOBT, or the fecal immunochemical test (FIT), is the most economical and easy-to-implement approach, so it has been recommended in several countries as a first-line population-screening approach [16, 63]. However, its accuracy is relatively lower and it must be followed by a colonoscopy to confirm the results [64]. A new method derived from FIT called multitarget stool DNA testing (DNA-FIT), which includes a series of hemoglobin and DNA mutations such as quantitative molecular assays for KRAS mutations, aberrant NDRG4, and BMP3 methylation. The FIT-DNA is more sensitive for detecting advanced precancerous lesions and CRC than FIT but has more false-positive results [65].

The colonoscopy, known as the golden standard for CRC screening and diagnosis, has high sensitivity and can perform excision for precancerous lesions, despite its higher costs. Thus, it can lower the incidence and mortality of CRC. After a 20-year follow-up of the US National Polyp Study cohort, CRC-specific mortality was ∼50% lower among subjects who at baseline had undergone endoscopic removal of adenomas than in an unscreened control cohort [66]. The results from a meta-analysis suggested a 40%–60% lower risk of incident CRC and death from CRC after screening colonoscopy [67].

Most European countries, the USA, and China have established CRC-screening guidelines and programs. Given the considerable rise in treatment costs, CRC screening is a cost-saving exercise in many countries. Most countries recommend FIT to those aged >50 years and with an average-risk population (China, Denmark, France, and Norway, etc.), while colonoscopy was only recommended in the USA, China, Germany, and Austria as a screening test [16]. As for frequency, most guidelines recommend that patients aged >50 years should take the FIT test once a year and once every 10 years for colonoscopy [16, 64, 68].

### Gastric cancer

Gastric cancer is estimated to be the fourth most common cancer in both sexes, and was the fourth leading cause of death among all cancer types worldwide in 2020 and the third most common cause of cancer death in 2018 [1, 4]. In 2020, it was estimated that there were 1.0 million (5.6%) new gastric-cancer cases and 0.7 million (7.7%) cancer deaths [4]. Gastric cancer used to be one of the major causes of cancer-related death, but the breakthrough in understanding the causation of stomach cancer, namely a bacterium—*Helicobacter pylori*—has successfully helped to reduce the incidence and mortality over the last century. However, patients with gastric cancer are often diagnosed with advanced disease and survival is poor.

### Epidemiology characteristics

**Figure 2.**
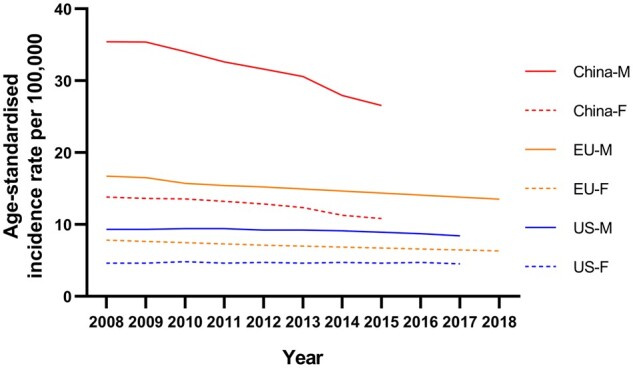
Time trends of incidence rates of gastric cancer in men and women across China, the USA, and Europe. Data from National Cancer Center (China), Centers for Disease Control and Prevention (the USA), and European Cancer Information System (EU).

Gastric cancer has very strong regional distribution differences, as Eastern Asia—China, Japan, and South Korea—contributes ∼65% of new global gastric-cancer cases each year [4]. In 2015, gastric cancer was the second most common cancer type in China (ASR 18.57 per 100,000) [12], whereas, in 2020, it was estimated to have been overtaken by CRC and became the third most common cancer [4]. Although its ASR is not the highest, China has the most gastric-cancer patients (∼478,000, 45% of new cases in 2020) all over the world [4]. Heterogeneities exist within European countries. Central and Eastern Europe have the second-highest incidence rate of gastric cancer in the world (ASR 11.3 per 100,000). On the contrary, the incidence is significantly lower in Northern and Western Europe (ASR 4.6–5.9 per 100,000), similar to that in the USA (ASR 4.2 per 100,000) [4]. The reasons for such differences are multiple and complex, and include genetic susceptibility, strains of *H. pylori*, hygiene, food preparation, and food preservation. Several migrant studies have shown that, after migration to low-incidence regions, the incidence for migrants was lowered for the first generation and was nearly similar to that for natives for the second generation, which suggests that environmental factors might be the main contributor to the development of gastric cancer [69]. Differently from CRC, improved living conditions associated with economic development have contributed to the reduction in incidence due to the clearance of *H. pylori* [11, 70]*.* In the USA and European countries, gastric cancer is expected to be a rare disease (defined as <6 per 100,000 person-years) by 2035, while the number of new cases will remain high and continue growing [71]. The incidence in China has also undergone a gradual decline in recent decades (Figure 2).

Gastric cancer is more common in men than in women (about 2:1) and the most common age at diagnosis is ∼60 years [72]. Of note, incidence increases were seen in younger age groups (<50 years) in both low- and high-incidence populations, especially in low-incidence countries such as the UK and the USA—populations with a typically low prevalence of *H. pylori* infection [73], which may imply that changes in the prevalence of some lifestyle factors have contributed to the increases shown in more recent generations [74]. Mortality, due to the implementation of the screening program, has had a substantial reduction in recent years [75–77]. In China, gastric cancer has been the third most common cause of cancer deaths for decades but it has shown a remarkable decline in mortality. In 1990–1992, the ASR for the mortality of gastric cancer was 40.8 per 100,000 for males and 18.6 per 100,000 for females [78], while, in 2015, it was down to 18.6 for males and 7.53 for females [12], and is expected to continue to decline in the future. However, due to population growth and aging populations, the number of deaths is expected to continuously grow.

### Risk factors

*Helicobacter pylori* infection is the most well-described risk factor for gastric cancer. Chronic infection of the gastric mucosa leads to stepwise progression from atrophic gastritis and intestinal metaplasia. Most *H. pylori* strains possess a cytotoxin-associated gene A (CagA) pathogenicity island—an oncoprotein that affects the expression of cellular signaling proteins [79]. Approximately 89% of non-cardia gastric cancers, representing ∼78% of all gastric cancers, can be attributable to *H. pylori* infection [80]. As estimated by a meta-analysis, there were ∼4.4 billion individuals with *H. pylori* infection worldwide in 2015 [70]. China had a prevalence estimate of 55.8% (95% CI: 51.8%–59.9%) and it was 35.6% (95% CI: 30.0%–41.1%) for the USA [70]. Consistently with the differences in the incidence rates in Europe, the prevalence estimates were highest in Eastern Europe (62.8%; 95% CI: 48.3%–77.2%) and lowest in Western Europe (34.3%; 95% CI: 31.3%–37.2%), and the prevalence estimates were 47.0% (95% CI: 41.8%–52.1%) for Europe as a whole [70]. In a study conducted in Japan, gastric cancer developed (over a mean follow-up of 7.8 years) in 2.9% of patients with peptic ulcer, dyspepsia, or gastric hyperplasia who had *H. pylori* infection, whereas no cases were detected in uninfected patients with these conditions [81]. In a Chinese cohort, the infection of *H. pylori* increased both cardia and non-cardia gastric-cancer risk compared to the non-infected population [82]. However, later studies have suggested that *H. pylori* infection is a risk factor only for non-cardia cancer and does not increase the risk of cardia cancer [83, 84]. As concluded by a meta-analysis, 5.9 is the best estimate of the relative risk of non-cardia cancer associated with *H. pylori* infection [84].

The Stomach Cancer Pooling Project, a consortium that included 23 epidemiological studies from Europe, North America, and Asia, found tobacco smoking was an important risk factor, no matter whether there was *H. pylori* infection or not [85]. Besides, the risk increased with the intensity and duration of smoking and decreased after smoking cessation. Other meta-analyses also concluded that smoking is the most important behavioral risk factor for gastric cancer, and increases the risk by ∼50% in males and 20% in females [86, 87]. Alcohol drinking is also recognized as a risk factor [88]. Drinking alcohol containing ethanol >10 g/day will increase the risk of gastric cancer and a dose–response meta-analysis showed a significant increase in risk in Asian males. Moreover, combined exposure to smoking and alcohol further increases the risk [87].

Foods preserved by salting, especially traditional Asian pickled foods, are considered to be associated with the development of gastric cancer. The consumption of foods preserved by salting increases the risk of gastric cancer. The WCRF/AICR found that people with a high intake of salt-preserved food had a 1.7-fold higher relative risk of gastric cancer compared to those with a low intake [88]. Another meta-analysis including 10 studies found that the population with the highest intake of salted vegetables had a 1.32 (95% CI: 1.10–1.58)-fold higher risk than those with the lowest intake [89]. Salted-food intake might increase the risk of *H. pylori* infection and could also act synergistically to promote the development of gastric cancer. Still, more research is needed to provide high-level evidence.

Obesity, which was not recognized as a risk factor for non-cardia gastric cancer, has a strong association with gastric cardia cancer [90]. As BMI increases by 5 kg/m^2^, the risk of gastric cardia cancer increases by 20%–30% [88, 91]. This finding corroborates the increasing obesity and numbers of gastric cardia cancers in the USA and Europe [2, 92, 93].

For other factors, such as low consumption of fruits and vegetables, results are mixed for both cardia and non-cardia gastric cancer [94–96]. For the consumption of processed meat, previous studies have shown that it was associated with an increased risk of gastric non-cardia cancer [97], but the results from newer cohort studies have shown that its effect was not significant [98, 99]. To reflect this contradiction, the WCRF/AICR have noted these factors as limited-suggestive [88].

### Primary prevention and screening

As gastric cancer is an infection-associated malignancy, the main part of the primary prevention of gastric cancer is eradicating *H. pylori*. Since oral–oral and fecal–oral routes have been postulated to be involved in the transmission of *H. pylori* [80], the first possibility for preventing the associated stomach cancer consists of avoiding infection through personal hygiene, control of water supplies, food-quality control, and other measures [100]. For now, the best approach for the diagnosis of *H. pylori* infection is 13 C-UBT (urea breath test), with high sensitivity and specificity, and excellent performance [101]. Using regimens that contain two or three generic antibiotics plus a proton-pump inhibitor for 7–14 days can achieve ∼80% success in eliminating *H. pylori* infection. However, until now, there have been limited data from randomized trials on the effects of eradicating *H. pylori.* Earlier results from Japan have shown that, after the endoscopic resection of early gastric cancer, the eradication of *H. pylori* statistically significantly reduced the risk of metachronous gastric cancer [102]. Another randomized trial in China found a statistically significant 39% reduction in gastric-cancer risk after *H. pylori* eradication [95]. A study in South Korea found that eradicating *H. pylori* after the endoscopic resection of gastric tumors lowered the incidence of metachronous gastric carcinoma, although this result was not statistically significant [103]. It can be concluded that *H. pylori* treatment could have lowered the gastric-cancer incidence by 30%–40% [104], but the available data do not permit precise estimation of the overall benefits and possible adverse consequences, such as increased esophagitis [105].

Screening for gastric cancer in the population includes two aspects: screening for precancerous lesions—upper-gastrointestinal series (UGI), serum pepsinogen testing (PG), and endoscopy; and screening for *H. pylori–H. pylori* serology [106].

The UGI was a standard method for diagnosing gastric diseases, but has a relatively low sensitivity of ∼38% [107]. Studies conducted in Japan showed that screening by UGI series resulted in an ∼40% reduction in gastric-cancer mortality [106], so UGI is still recommended in the national gastric-cancer screening programs in Japan and Korea. PG testing is receiving wide recognition in Japan and China owing to its convenience, freedom from discomfort or risk, efficiency, and economy. PG I ≤ 70 ng/L and PG I/II ratio ≤ 3.0 are associated with an increased risk of gastric cancer [108]. In a meta-analysis assessing ∼300,000 people, the sensitivity and specificity of PG testing for gastric-cancer screening were 77% and 73%, respectively [109]. Endoscopy, which is the criterion for the diagnosis of gastric cancer, is the only method for direct visual examination of the gastric mucosa and it allows biopsy sampling so that histologic evaluation can be performed. Endoscopy is the primary method for gastric-cancer screening in Japan and South Korea, and is also highly recommended in China [110]. Endoscopy was reported with a sensitivity of 88%–95% and specificity of 85%–88% [106, 111]. A nested case–control study from Korea reported a 47% reduction in mortality from gastric cancer by using endoscopic screening [75].

*Helicobacter pylori* serology is used to detect the antibody of *H. pylori*, and the presence of *H. pylori* antibody is associated with the presence of gastric cancer or precancerous lesions [112]. Serology is cheap compared to other non-invasive test approaches; hence, serology was felt to be the best current option in the Asia–Pacific region for a population-based screening approach [113]. However, due to its low sensitivity and specificity, it is recommended to be combined with other screening approaches.

Right now, only Japan and South Korea have a comprehensive screening system for gastric cancer. To reflect recent studies and epidemiology reports, in 2015, the Japanese gastric screening guideline as adjusted the starting age of screening to 50 years and the screening interval to 2–3 years [106]. In Korea, people >40 years old receive either a UGI or endoscopy test for gastric-cancer screening every 2 years [114]. Chinese experts’ consensus on gastric-cancer screening recommends that people >40 years old and with at least one risk factor (with *H. pylori* infection, in a high-incidence-rate region, with a family history, with precancerous diseases, with other lifestyle risk factors) to start the serological biopsy for five biomarkers (PGI, PGII, PGI/PGII ratio, *H. pylori* antibody, and gastrin-17) as the first step for screening and stratifying [112]. By using risk stratification, patients with different risks underwent different screening projects based on endoscopy.

### Esophageal cancer

Esophageal cancer ranks eighth in terms of incidence (604,100 new cases, 3.1%) and sixth in mortality (544,076 new deaths, 5.5%) overall [4]. The two histological types of esophageal cancer—esophageal squamous cell carcinoma (ESCC) and esophageal adenocarcinoma (EAC)—differ in populations and have completely distinct biological characteristics, geographical distributions, risk factors, and temporal trends [115]. Patients with either cancer are both diagnosed at an advanced stage due to the late occurrence of symptoms. Thus, the prognosis is usually poor, at ∼5%–34% [116]. Therefore, identifying its distributional characteristic and risk factors and promoting practical and accurate prevention and screening methods are crucial to reducing the global burden of esophageal cancer.

### Epidemiology characteristics

The highest incidence rate of esophageal cancer can be found in Eastern Asia, with an ASR of 12.3 per 100,000 in 2020 [4]. However, esophageal cancers, both ESCC and EAC, are not a common cancer type in Europe and America (Table 1). Furthermore, the predominant histological type also varies from East to West [115].

**Figure 3.**
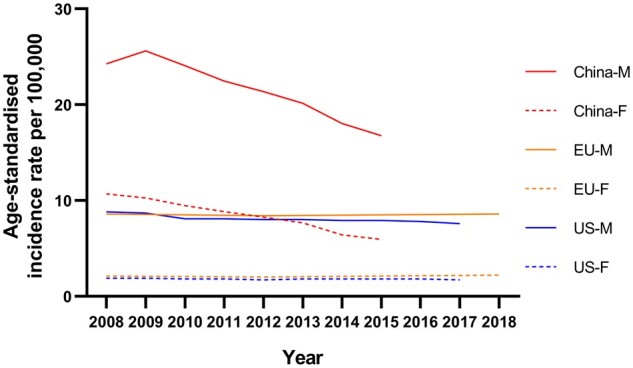
Time trends of incidence rates of esophageal cancer in men and women across China, the USA, and Europe. Data from National Cancer Center (China), Centers for Disease Control and Prevention (the USA), and European Cancer Information System (EU).

ESCC comprises ∼90% of all cases [117]. ESCC has significant geographical-distribution differences. China has about half of all esophageal-cancer cases around the world, among which most cases are ESCC. In 2015, esophageal cancer was the sixth most common cancer in China, with ∼246,000 new cases (ASR 11.3 per 100,000), and it was estimated to have reached 13.8 per 100,000 in 2020 [4, 12]. The incidence rates for esophageal cancer in different regions can vary by ∼10-fold within China. Most of the ESCC cases are in the North Central region, especially the area around the Taihang Mountain, where the ASR reaches 80–110 per 100,000 in males and 40 per 100,000 in females [118], and Cixian, which is the most studied area for esophageal cancer in China, with the highest ASR in both males and females in the world (192.7 per 100,000 in males; 108.5 per 100,000 in females) [119]. ESCC has a relatively obvious sex difference. The incidence rate in men is three times that of women, which could be partly attributed to the unbalanced distribution of risk factors—the use of tobacco and alcohol between men and women. Because of the bad prognosis, mortality is relatively high. In 2015, esophageal cancer was the fourth most common cause of cancer death in China (188,000 deaths, ASR 8.36 per 100,000) [12]. Due to the implementation of screening, the control of risk factors in a large population, and advances in the clinical management of ESCC, the incidence and mortality of ESCC have been reduced in recent decades in China [3, 11, 120, 121] (Figure 3) and are predicted to continue decreasing in the coming years [122].

In 2012, an estimated 52,000 individuals (41,000 men and 11,000 women) developed EAC worldwide, resulting in a global incidence rate of 0.7 per 100,000 person-years (1.1 in men and 0.3 in women) [115]. Differently from ESCC, 53% of patients were from Europe, Northern America, or Oceania. The incidence of EAC has increased in many Western countries in recent decades [123]. Moreover, in some countries, including the UK and the USA, EAC has surpassed ESCC and become the predominant histological type of esophageal cancer [124]. It is expected to rise dramatically across high-income countries and will displace ESCC in more countries, such as Italy, Spain, and France, in the coming years [122]. Similar to ESCC, EAC also shows a striking male predominance in incidence. The highest sex difference can be found in the USA, as the males/females incidence ratio is 9:1 [115, 125].

### Risk factors

ESCC and EAC have different etiological risk factors [116], which also reflect their difference in pathogenesis [126]. The pathophysiological pathway of ESCC is typically initiated by carcinogenic compounds in direct contact with the esophageal mucosa and thereby leads to esophageal squamous dysplasia. It is now widely recognized that smoking and alcohol overconsumption are both risk factors for ESCC [127], and the risk is higher when they are in combination [128]. Ever smokers had significantly higher risks of ESCC, with an OR of 2.8 as reported by a population-based case–control study [129]. An intensity-duration cumulative exposure effect was demonstrated by a study that, for equal pack-years, the mild intensity of smoking for a long time has a higher risk than stronger intensity for a shorter time [130]. Dose–response meta-analyses including six studies found a relative risk of 1.25 (95% CI: 1.12–1.41) per 10 g/day ethanol intake [127]. Another pooled analysis found that, when compared with no drinks, at least seven drinks per day had a relative risk of 9.62 (95% CI: 4.26–21.71) [131].

There are some other factors reported to be associated with ESCC, but at a relatively lower level of evidence, including the consumption of vegetables and fruits and processed meat. Most studies concluded that a higher intake of fruits and vegetables probably decreases the risk of ESCC [132]. One reported that 100 g/day of vegetable intake could reduce the risk of ESCC by 16%, but no significance was found between the highest vs lowest intakes [133]. This meta-analysis also reported that a 100-g/day consumption of fruits could reduce the risk by 39%, but with high heterogeneity (*I*^2^ = 90%). Regarding processed meat, which is often listed as a risk factor for GI cancers, studies found a 41% increase in risk in the highest-consumption population [134], but another meta-analysis found no significance [135]. Thus, the WCRF/AICR marked these factors as having limited evidence. Other factors, such as high-temperature drinks [136], HPV infection [137], and BMI [138], were reported to be related to ESCC, but the literature did not draw a consistent conclusion.

Differently from ESCC, alcohol was found not to be associated with EAC [131, 139], and the effect of smoking is also weaker than that in ESCC [127, 129], although it is still a strong risk factor for EAC [140, 141]. Furthermore, obesity and gastroesophageal reflux disease (GERD) are the distinct risk factors for EAC. Increasing BMI has been consistently associated with increased risk of EAC in a seemingly linear exposure–response pattern, with a relative risk of 2.7 (95% CI: 2.2–3.5) for patients with a BMI ≥ 30 kg/m^2^ [142, 143]. Similarly to CRC, predominantly central and intra-abdominal adiposity has a bigger influence than BMI alone [90, 144]. Obesity may explain partially the increase in EAC incidence in Western countries, especially in white people. Moreover, adiposity causes increased intra-abdominal pressure and facilitates reflux [145], which has been proven to be another important risk factor. GERD is a strong and dose-dependent risk factor for EAC, as confirmed in population-based studies [142, 146]. Results from a meta-analysis have shown that daily symptoms of reflux increased the odds of EAC by >7-fold [147]. Continuous GERD leads to the development of Barrett’s esophagus, which presents as metaplasia of the distal esophageal mucosa and is characterized as the precursor to EAC [148]. According to a population-based cohort study in Norway, weekly symptoms of GERD increased by ≥47% during 1995–2006 [149], which corresponded to the rapid increase in EAC incidence during this decade [115]. Interestingly, in contrast to its effects on gastric cancer, *H. pylori* infection may reduce the risk of EAC [150]. This phenomenon may be due to the gastric atrophy induced by *H. pylori* infection, which contributes to lesser gastric acid reflux and thereby lowers the risk [151]. Since the infection rate is continuously decreasing in Western countries, the effects of *H. pylori* on EAC and the individual eradication strategies need more thorough studies to confirm [152].

### Prevention and screening

When the symptoms of both types of esophageal cancer start to surface, the cancer is often at an advanced stage and has a very poor prognosis, emphasizing the significance of prevention and early detection to lower the burden of esophageal cancer.

The proton-pump inhibitors (PPIs) [153, 154] and NSAID/aspirin [155] have shown protective effects for esophageal cancer. The PPIs were reported to decrease the risk of dysplasia and adenocarcinoma in patients with Barrett’s esophagus [156]. However, as the results were concluded from the observation of patients with GERD and Barrett’s esophagus after anti-reflux surgery, no reduction in the risk of EAC was found [157]. Thus, the PPIs are still not recommended conventionally for cancer prevention. The NSAID/aspirin was shown to reduce the risk of ESCC and EAC by 30%–40% [158]. However, results from a randomized trial showed that celecoxib did not affect the progression of both esophageal squamous dysplasia and Barrett's dysplasia [159, 160]. Given the additional preventive benefits of the use of aspirin for other cancer types and cardiovascular disease, these drugs may be good candidates for chemoprevention in groups at high risk [161].

Detection of esophageal cancer at an earlier, potentially curable stage is crucial to improving patient survival. Now, endoscopy is widely accepted as the best method for esophageal screening and diagnosis. Endoscopic screening for precursor lesions and endoscopic resection or ablation of the dysplastic lesions have been shown to reduce the risk of developing ESCC and dying from the disease [121]. For EAC, the current British Society of Gastroenterology and American College of Gastroenterology guidelines suggest screening in patients who have a history of GERD lasting >5 years and have multiple other risk factors, including male sex, Caucasian race, central obesity, and current or past history of smoking [162, 163]. For ESCC, there is currently no guideline for screening, but Chinese experts drafted a consensus on esophageal-cancer screening in 2014 [164]. The consensus recommends that people >40 years old and with at least one risk factor (from an esophageal-cancer-prevalent region, with upper-GI symptoms, with an esophageal familial history, with precursor diseases of esophageal cancer, with other high-risk factors) should undergo endoscopic screening. The following screening or treatment plans are decided based on the results of endoscopy and biopsy.

However, endoscopy is invasive and expensive, and, despite the rapid increase in incidence in recent decades, the low absolute numbers of esophageal-cancer cases in the West remain a barrier to the implementation of screening programs. Some other non-endoscopic screening methods have emerged in recent years and reported their preliminary results. For ESCC, the capsule-sponge methodology had a sensitivity and specificity of 100% and 97%, without extra safety problems and unsatisfied experience [165]. By using a minimally invasive cell-sampling device and immunohistochemical staining for Trefoil Factor 3, the sensitivity and specificity reached 87.2% and 92.4% in diagnosing patients with ≥3 cm of circumferential Barrett’s esophagus [166].

## Discussion and conclusion

As concluded in Table 2, an unhealthy lifestyle, including overuse of alcohol and smoking, is the main risk factor for all GI cancers and, because of their direct contact with food, the dietary pattern is also highly associated with all GI cancers. Thus, primary prevention of GI cancer is the most efficient and cost-beneficial means of reducing the cancer burden. A healthy lifestyle can lower the risk of all GI cancers.

**Table 2.** Characteristics of each gastrointestinal (GI) cancer

GI cancers	Precancerous lesions	Environmental factors of strong level of evidence	Environmental factors of moderate level of evidence	Environmental factors of limited level of evidence	Primary prevention	Screening methods	Guidelines and screening programs
Colorectal cancer	Adenomas and serrated polyps	Obesity (unfavor) Processed meat (unfavor) Alcohol (unfavor) Smoking (unfavor) Physical activity (favor)	Read meat (unfavor) Dietary fiber (favor) Wholegrains (favor) Calcium intake (favor)	Vegetable and fruits (favor) Vitamin C (favor) Vitamin D (favor)	Risk-factor controls Aspirin	FOBT/FIT DNA-FIT Colonoscopy	USA, UK China Japan Germany Australia etc.
Gastric cancer	Atrophic gastritis and intestinal metaplasia	*H. pylori* infection (unfavor) Smoking (unfavor)	Alcohol (unfavor) Obesity (cardia) (unfavor) Salt-preserved food (unfavor)	Processed meat (non-cardia) (unfavor) Fruits (favor) Fiber (favor), Vegetables (favor)	Risk-factor controls Eradication of *H. pylori* NSAIDs/aspirin	Upper-GI series Pepsinogen, *H. pylori* serology Endoscopy	Japan South Korea
Esophageal squamous cell carcinoma	Esophageal squamous dysplasia	Alcohol (unfavor) Smoking (unfavor)	–	Processed meat (unfavor) Vegetables and fruits (favor) Physical activity (favor) HPV infection (unfavor)	Risk-factor controls NSAIDs/aspirin	Endoscopy	–
Esophageal adenocarcinoma	Barrett’s esophagus	Obesity (unfavor) GERD (unfavor) Smoking (unfavor)	–	Vegetables (favor) Physical activity (favor) Fiber (favor)	Risk-factor controls PPIs NSAIDs/aspirin	Endoscopy	USA UK

FOBT, fecal occult blood test; FIT, fecal immunochemical test; NSAIDs, non-steroidal anti-inflammatory drugs; GERD, gastroesophageal reflux disease; PPIs, proton-pump inhibitors.

These GI cancers are also similar in that they all have identified precursor diseases: adenomas and serrated polyps for CRC, atrophic gastritis and intestinal metaplasia caused by *H. pylori* infection for gastric cancer, and esophageal squamous dysplasia/Barrett’s esophagus for esophageal cancer. Thus, GI cancers can be diagnosed in a precancerous state and early treatment can reduce both incidence and mortality. However, there are only guidelines and screening programs for CRC and gastric cancer because of the geographical-distribution differences of cancers and the lack of cheap and relatively reliable approaches like FIT in CRC. Thus, future orientation will be the development of screening methods with both reliability and practicality, and the ability to recognize specific patients at high risk of GI cancer to perform personalized screening.

GI cancers are a major medical and economic burden worldwide. The last century has witnessed that, regardless of the level of economics, medical conditions, or public health, there is always a prevalent GI cancer that changes accordingly. Esophageal and gastric cancers are most common in developing countries, while CRC is the predominant GI malignancy in developed countries. In this article, we have reviewed the epidemiology, risk factors, prevention, and screening of these three cancers in China, the USA, and Europe. They share some common features, but also have distinct characteristics that imply the differences in population susceptibility and pathways to development malignancies. With the establishment of screening guidelines and the implantation of screening programs in more and more countries, the incidence and mortality rate of GI cancers are expected to decline in the future. However, due to the continuously increasing exposure to some risk factors such as obesity and the aging of the population, GI cancers will still be a great global health burden for a long time.

## Authors’ Contributions

Y.M.X. drafted the manuscript; L.S. collected and analysed the data; X.S.H. conceived of the project and revised the manuscript; Y.X.L. revised the manuscript and worked on the final approval of the version to be published.

## Funding

This work was supported by Sun Yat-Sen University Clinical Research 5010 Program [No. 2018026, YL] and the ‘Five Five’ Constructive Talent Project of the Sixth Affiliated Hospital of Sun Yat-Sen University [No. P20150227202010244, JW; No. P20150227202010251, YL].
